# Structure and Dynamics of Water Confined in Transition Metal Carbide MXenes: Implications for Electrochemical Applications

**DOI:** 10.1021/acsanm.5c05156

**Published:** 2026-01-29

**Authors:** Kaitlyn Prenger, Alexander I. Kolesnikov, Naresh C. Osti, Eugene Mamontov, Jong K. Keum, Kenneth C. Littrell, Michael Naguib

**Affiliations:** † Department of Physics and Engineering Physics, Tulane University, New Orleans, Louisiana 70118, United States; ‡ Neutron Scattering Division, 6146 Oak Ridge National Laboratory, Oak Ridge, Tennessee 37831-6473, United States; § Center for Nanophase Materials Sciences, 6146 Oak Ridge National Laboratory, Oak Ridge, Tennessee 37831, United States; ∥ Neutron Technologies Division, 6146 Oak Ridge National Laboratory, Oak Ridge, Tennessee 37831, United States

**Keywords:** MXenes, two-dimensional carbides, neutron scattering, confined water, water diffusion

## Abstract

Two-dimensional transition metal carbides and nitrides (MXenes) are an important family of electrochemically active 2D materials. MXenes combine high conductivity with hydrophilicity, making them attractive materials for many applications, including electrochemical energy storage, sensing, desalination, and others. In order to better understand the role of structure on MXene properties, here, we investigated the vibrational properties and diffusion of water in MXenes with differing layer thicknesses and transition metal compositions using inelastic, quasi-elastic, and small-angle neutron scattering. We found that all of the Mo-containing MXenes studied here exhibited comparable vibrational dynamics and diffusion coefficients to each other and to previously studied Ti_3_C_2_T_*x*_. However, Ti_2_CT_*x*_ was distinguished by its faster diffusion and more hydroxyl groups compared to the other MXenes studied. These results can help guide the selection of appropriate MXenes for energy storage and electrochemical water purification applications.

## Graphical abstract

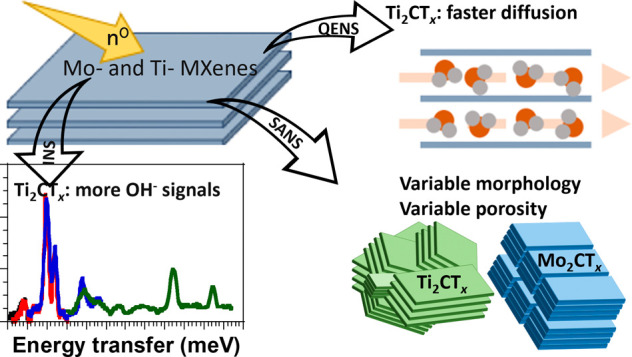

## Introduction

First described in 2011, MXenes are a family of two-dimensional (2D) transition metal carbides and carbonitrides.^1,2^ Generally synthesized from the large family of MAX phase ceramics,^3^ the number of MXenes described to date exceeds 50,^4,5^ with many more theorized as stable structures.^6^ MXenes are produced by selectively etching the A layer from the layered MAX ceramics, yielding layered materials with the composition of M_*n*+1_X_*n*_T_*x*_, where M is an early transition metal, X is carbon and/or nitrogen, and *n* = 1, 2, 3, or 4 and T_*x*_ stands for surface terminations (e.g., O, OH, and F).^1,7–9^ MXenes have already been shown to be promising for many applications, such as in ion batteries and supercapacitors, sensors, and in water purification.^1,7,8,10^

The surfaces of as-synthesized MXenes are terminated with −O, −F, and −OH groups, which makes them hydrophilic.^2,11^ MXenes combine this with electrical conductivity, which makes them an excellent material for use in aqueous supercapacitors. Water intercalation between the MXene layers is predicted to have a large impact on the electrochemical and structural properties of MXene.^12^ A recent study showing a link between the thermal dependence of electrical resistivity and the dynamics of confined water only serves to emphasize the importance of understanding the nature of water confined in MXenes.^13^

Inelastic neutron scattering (INS) has proven itself to be a powerful technique to study the vibrational dynamics for surface terminations in MXenes and intercalated species between the layers.^14–16^ For example, INS showed that hydrazine treatment of Ti_3_C_2_T_*x*_ reduces the amount of intercalated water, as well as reducing the number of −OH and −F terminations.^14^ This study was then used to explain the behavior of MXene microsupercapacitors in the presence of hydrazine.^17^ Osti et al. showed that Ti_3_C_2_T_*x*_ MXene annealed under vacuum to remove bulk water showed evidence of intercalated molecular hydrogen when the etching was carried out with 48% hydrofluoric acid (HF), but not when 10% HF was used.^18^ A follow-up work on pristine Ti_3_C_2_T_*x*_ MXene and cation intercalated Ti_3_C_2_T_*x*_ MXene showed that this molecular hydrogen was not present in intercalated materials, and that intercalated MXenes had less confined water.^19^

Quasi-elastic neutron scattering (QENS) has been shown to be a useful technique to examine the dynamics of water trapped in MXenes and diffusion coefficients. Osti et al. used QENS in conjunction with other techniques and modeling to examine Ti_3_C_2_T_*x*_ and potassium (K^+^)-intercalated Ti_3_C_2_T_*x*_ and showed the differences in diffusion coefficients between the intercalated and nonintercalated materials.^18^ These results were used to explain the choice of Ti_3_C_2_T_*x*_ for uranium removal and to understand the interlayer spacing changes in Ti_2_CT_*x*_ for thorium removal.^20,21^ They were further used to understand capacity loss in V_2_CT_*x*_ batteries.^22^ A related study on K^+^ and magnesium (Mg^+^) intercalated MXenes also showed that water resided between MXene layers, and that intercalation with potassium reduced water mobility while intercalation with magnesium increased it.^23^ The results of this study were used to guide the development of Ti_3_C_2_T_*x*_ microsupercapacitors.^24^ These two INS/QENS studies on intercalation were used to help explain the differences in capacitance of cation intercalated Ti_3_C_2_T_*x*_.^25^ QENS was also used to compare water dynamics in Ti_3_C_2_T_*x*_ with Ti_3_CNT_*x*_, helping to confirm the lack of −OH terminations in the latter.^26^ More recently, QENS was used on pristine and porous Ti_3_C_2_T_*x*_ at temperatures between 20 and 300 K to investigate the activation energy and dynamic modes of trapped water and hydrogen-containing surface groups.^27^ Another recent study employed QENS to understand why a 0.8:0.2 ratio of ionic liquid to salt in a protic ionic liquid electrolyte was the optimal composition, where QENS showed that particle mobility of the confined electrolyte increased at the optimized ratio compared to other ratios employed.^28^

MXenes have not been studied extensively with small-angle neutron scattering (SANS) as compared to other neutron scattering techniques, but SANS is another important technique to examine nanostructural characteristics. SANS was used along with QENS on Ti_3_C_2_T_*x*_ and potassium-intercalated Ti_3_C_2_T_*x*_, which suggested the presence of relatively rough surfaces for both materials, but the length scales were not compatible with the *Q* range studied to yield more conclusive analysis.^18^ The results of this study could be extrapolated to copper intercalation for water purification.^29^ A more recent study on delaminated Ti_3_C_2_T_*x*_ in D_2_O used SANS to confirm a lamella model for the material and extract dimensional parameters.^30^ These findings helped to explain the nanostructure of a NiMo_3_S_4_-MXene hybrid used as an HER catalyst.^31^

These neutron scattering techniques do have the potential to guide the selection of materials and the modification of those materials for energy storage applications. Since MXene has excellent performance in aqueous supercapacitors, understanding the water diffusion and protic species on MXene surfaces is key to understanding the nature of the underlying reactions driving the capacitance.^25,32,33^ Further, because even annealed MXene retains some water on the surfaces, understanding this water is key to understanding how it will interact with organic and ionic liquid electrolytes.^22,34,35^ Additionally, the use of MXenes for water purification has also been well documented in the literature, with several water purification studies already using the results of neutron studies to understand their systems.^20,21,29^

Neutron scattering techniques have the potential to be a useful tool for investigating MXenes, but to date nearly all neutron investigations have been done on the Ti_3_C_2_T_*x*_ system,^14,18,19,23,28,36,37^ with only one study comparing that MXene to its corresponding carbonitride.^26^ The family of MXenes is much larger than this one representative, however. All MXenes have the same basic layered hexagonal structure, but changing either the transition metal or the value of *n* can have a strong effect on properties.^5,7^ Even when comparing MXenes with the same transition metal and similar surface chemistry but different *n*, we find that Ti_3_C_2_T_*x*_ and Ti_2_CT_*x*_ show different electrochemical performance in lithium ion batteries, with Ti_2_CT_*x*_ having approximately 1.5 times higher gravimetric capacity, since ions intercalate between MXene layers, rather than within them.^33,38,39^ Thus, it is important to study the structure and dynamics of MXenes beyond Ti_3_C_2_T_*x*_.

Here, we investigate the effect of different structures (*n* = 1, 2, or 3) and different transition metals on confined water, surface hydrogen, and surface −OH groups through the use of INS, QENS, SANS, and SEM. SANS, XRD, and SEM were used to characterize the sample structure on different length scales, and INS and QENS were used to measure vibrational and diffusional behavior of water molecules in the samples, respectively. SANS (*Q* = 0.0006 to 1 Å^–1^) is used to characterize the sample structure on a large scale (*d* = 2π/*Q*, *d* ≈ 1 to 1000 Å), and XRD is used for the scale comparable to interatomic distances (λ = 1.54 Å, 2θ = 3 to 65°, *d* ≈ 1.5 to 30 Å). For these studies, we used a series of MXenes, namely, Ti_2_CT_*x*_, Mo_2_CT_*x*_, Mo_2_TiC_2_T_*x*_, and Mo_2_Ti_2_C_3_T_*x*_. The Mo–Ti system, which has members with *n* = 1, 2, or 3: Mo_2_CT_*x*_, Mo_2_TiC_2_T_*x*_, and Mo_2_Ti_2_C_3_T*_x_*, allows for the probing of the effect of layer thickness.^40–43^ This system also possesses ordering in the transition metal layer, with molybdenum layers surrounding the titanium layer/layers.^40–42^ The inclusion of Ti_2_CT_*x*_ allows for a direct comparison with Mo_2_CT_*x*_—both have *n* = 1—as well as to the existing Ti_3_C_2_T_*x*_ literature, since both are Ti-based MXenes.

While the results from all of the Mo-containing MXenes align with previous measurements of Ti_3_C_2_T_*x*_, Ti_2_CT_*x*_ shows some marked differences from other MXenes studied using neutron scattering. Strikingly, there is much more mobile water in Ti_2_CT_*x*_, including inelastic signatures of mobile water that are not found in other MXenes. QENS of Ti_2_CT_*x*_ needed to be fit with a two-component model and had much faster diffusion compared to other MXenes studied. Finally, SANS suggests that Ti_2_CT_*x*_ has increased roughness and more slit pores in the longer-range structure compared to those of the Mo-containing MXenes.

## Materials and Methods

### Sample Synthesis

Ti_2_AlC, Mo_2_TiAlC_2_,^41^ and Mo_2_Ti_2_AlC_3_ ^44^ were synthesized by mixing molybdenum (Mo, −325 mesh, 99.9%, Alfa Aesar) and/or titanium (Ti, −325 mesh, 99.9%, Alfa Aesar), aluminum (Al, −325 mesh, 99.9%, Alfa Aesar), and carbon (C, APS 7–11 μm, 99%, Alfa Aesar) powders with atomic ratio of 2.0:1.1:1.0 (Ti:Al:C for Ti_2_AlC), 2.0:1.0:1.2:2.0 (Mo:Ti:Al:C for Mo_2_TiAlC_2_) or 2.0:2.0:1.3:2.7 (Mo:Ti:Al:C for Mo_2_Ti_2_AlC_3_) in a Turbula T2F mixer for 3 h at 56 rpm. Yttria-stabilized zirconia balls (10 mm) were used as mixing media. Then, the mixed powders were heated at a heating rate of 10 °C/min to 1400 °C (for Ti_2_AlC) and held at that temperature for 1 h, or to 1600 °C (for Mo_2_TiAlC_2_ and Mo_2_Ti_2_AlC_3_) and held at that temperature for 4 h in a tube furnace with continuously flowing argon (Ar). Mo_2_Ga_2_C^45^ was prepared by mixing liquid gallium (99.5%, Alfa Aesar) with Mo_2_C (−325 mesh, 99.5%, Alfa Aesar) in an atomic ratio of 8:1. The mixture was heated to 850 °C at a rate of 10 °C·min^–1^ in a tube furnace with flowing Ar, held for 12 h, and then allowed to cool to room temperature. The mixture was stirred to further incorporate the Ga and Mo_2_C, and then the heating cycle was repeated. The resulting mixture was added to 37% HCl (Fisher) in a ratio of 1 g of the mixture:20 mL of the acidic solution, and stirred for 72 h to dissolve excess Ga.

To synthesize Ti_2_CT_*x*_ MXene, Ti_2_AlC was ground to −200 to +325 mesh and then soaked in 10% hydrofluoric acid (HF, 48–51%, Sigma-Aldrich) with a ratio of 10 mL liquid: 1 g MAX for 2 h with stirring at room temperature. The Mo-containing MAX was ground to −400 mesh and then added to hydrofluoric acid. Mo_2_TiAlC_2_ and Mo_2_Ti_2_AlC_3_ were added in a ratio of 1 g: 10 mL 50% hydrofluoric acid and held at 55 °C for 90 h, then the acid was replenished, and they were allowed to etch for another 48 h. Mo_2_Ga_2_C was added to 14 M HF at a ratio of 1 g: 40 mL acid and held at 55 °C with stirring for 160 h. At the end of the etching period, all mixtures were washed with copious portions of deionized (DI) water until a pH of >6 was reached and then dried using vacuum-assisted filtration, resulting in a cake of MXene powders. To form the K-intercalated Mo_2_TiC_2_T_*x*_, 2 g of the etched MXene was soaked in 1 M KCl for 24 h, then washed 3 times with 50 mL of deionized water, followed by vacuum-assisted filtration. To prevent oxidation of samples, all samples were stored in an Ar-filled glovebox until synthesis was complete and ready to be annealed and packed into sample cans. Approximately 1.5 g of each MXene powder was annealed under vacuum at 110 °C for 4 h to remove bulk water. The samples were immediately transferred to an Ar-filled glovebox, and approximately 1 g of each annealed MXene was placed in a flat plate aluminum sample holder (50 × 50 mm^2^ cross-section) and sealed with an indium wire gasket for neutron scattering measurements.

### X-ray Diffraction and Microscopy

X-ray diffraction (XRD) patterns were collected for the MAX powders and MXenes before and after annealing using a Rigaku DMax 2200 diffractometer using a Cu K_α_ radiation source. These scans were conducted with a step size of 0.02° and a 1° per minute scan rate from 3.0 to 65.0° 2θ. Scanning electron microscopy (SEM) images were taken using a Hitachi 4800 high-resolution SEM. To confirm full etching, energy-dispersive X-ray spectroscopy was performed on a Hitachi 3400 electron microscope with Oxford Inca software.

### Inelastic Neutron Scattering

INS measurements were made using the Fine Resolution Fermi Chopper Spectrometer (SEQUOIA)^46^ at Oak Ridge National Laboratory’s Spallation Neutron Source (SNS). The MXene samples and an empty sample plate were cooled to 5 K, and then INS measurements were made at four incident energies (*E*_*i*_) of 55, 160, 250, and 600 meV, to provide high energy resolution (about 1–3% of *E*_*i*_) in a wide range of energy transfer. The empty container measurements were used to subtract background data from the sample measurements. The details of the INS data analysis can be found in the Supporting Information.

### Quasi-Elastic Neutron Scattering (QENS)

QENS experiments were carried out on the backscattering silicon spectrometer (BASIS)^47^ at the SNS of Oak Ridge National Laboratory, Oak Ridge, Tennessee, USA. BASIS provides a fine energy resolution of 3.7 μeV (full width at half-maximum) while operating the instrument in the standard configuration (incoming neutrons with bandwidth centered at 6.4 Å, choppers spinning at 60 Hz, and utilizing Si 111 analyzer panels). In this configuration, an energy transfer range of ± 100 μeV and a Q (momentum transfer vector) range of 0.2–2.0 Å^–1^ can be accessed. QENS spectra from each sample, loaded into a flat plate aluminum can with an insert providing 0.25 mm thick sample (the same samples and cans as were used for INS measurements), were collected at 20 K (for an instrument resolution) and 300 K. Neutron detector efficiency was accounted for by normalizing the spectrum collected from the sample using the data obtained from an empty vanadium can, a process commonly referred to as vanadium normalization. Vanadium normalization and background (scattering from an empty can) subtraction were done during the data reduction using MantidPlot.^48^ The DAVE software package was used to perform the data analysis. For details of the QENS data analysis, please see the Supporting Information.

### Small-Angle Neutron Scattering

The SANS data were collected using the CG-2 General-Purpose SANS instrument^49^ at the High Flux Isotope Reactor of Oak Ridge National Laboratory in 3 different instrument configurations-- sample-to-detector distance of 19.2 m, source aperture-to-sample distance of 17.3 m, and central wavelength of 19 Å; sample-to-detector distance of 7.0 m, source aperture-to-sample distance of 7.2 m; and central wavelength of 4.75 Å, and sample-to-detector distance of 1.0 m, source aperture-to-sample distance of 7.2 m, and central wavelength of 4.75 Å, all with the 1 m square area detector laterally offset by 0.4 m to maximize the Q range measured in each setting to span a total range of 0.0006 < Q < 1 Å^–1^. The data were corrected for detector pixel position nonlinearity, area, and efficiency, and empty- and blocked-beam scattering, placed on an absolute scale using comparison to the attenuated direct beam, and azimuthally averaged using the standard procedure and reduction codes provided by the instrument scientist. Details of the SANS data analysis can be found in the Supporting Information.

## Results and Discussion

A representative schematic of the etching process is shown in Figure 1. Basic characterization (i.e., XRD and SEM) of the etched MXenes confirmed that the target MXenes were produced. XRD patterns of pre- and postannealing (at 110 °C for 4 h) MXenes are presented in Figure 2a. All MXenes show a shift to a lower *c*-lattice parameter (*c*-LP) after annealing, which is expected due to the partial removal of intercalated water during annealing. Previous studies had shown a decrease in the *c*-LP after annealing in Ti_3_C_2_T_*x*_, from 19.88 Å to 19.62 Å.^19^ Mo_2_Ti_2_C_3_T_*x*_ shows the largest change, dropping from 32.05 to 29.82 Å, a difference of 2.23 Å. Mo_2_CT_*x*_ and Mo_2_TiC_2_T_*x*_ had similar shifts of <1 Å, with Mo_2_CT_*x*_ shifting from 20.87 Å to 19.91 Å and Mo_2_TiC_2_T_*x*_ shifting from 25.74 Å to 24.80 Å. Before annealing, Mo_2_TiC_2_T_*x*_ intercalated with K^+^ had a slightly lower *c*-LP than the nonintercalated sample, with 25.62 Å, but demonstrated the smallest shift after annealing, shifting to 25.10 Å, a difference of only 0.52 Å. This agrees with previous work on K^+^ intercalated Ti_3_C_2_T_*x*_, which showed that the *c*-LP remains relatively constant when intercalated with K^+^.^23,25,50–52^ The Ti_2_CT_*x*_ *c*-LP also has a small shift from 25.23 Å before annealing to 24.53 Å afterward. Considering that both Mo_2_CT_*x*_ and Ti_2_CT_*x*_ have the same *n*, such a large difference in the *c*-LP between the two MXenes suggests the presence of more intercalants or weaker hydrogen bonding in the case of Ti_2_CT_*x*_ with a large *c*-LP.

**Figure 1:**
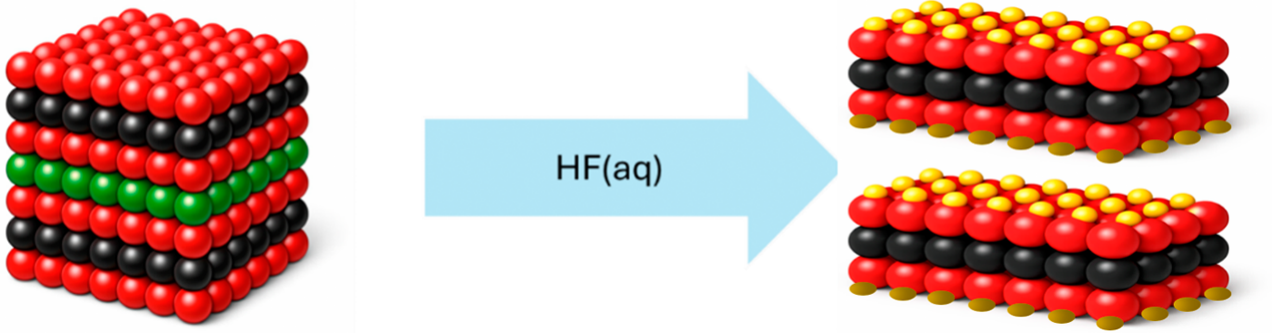
Etching of the MXene. Material shown is Ti_2_AlC etched to form Ti_2_CT_*x*_. Red balls represent titanium, black balls represent carbon, and green balls represent aluminum. Surface terminations (−O, −OH, and – F) are represented by yellow.

**Figure 2:**
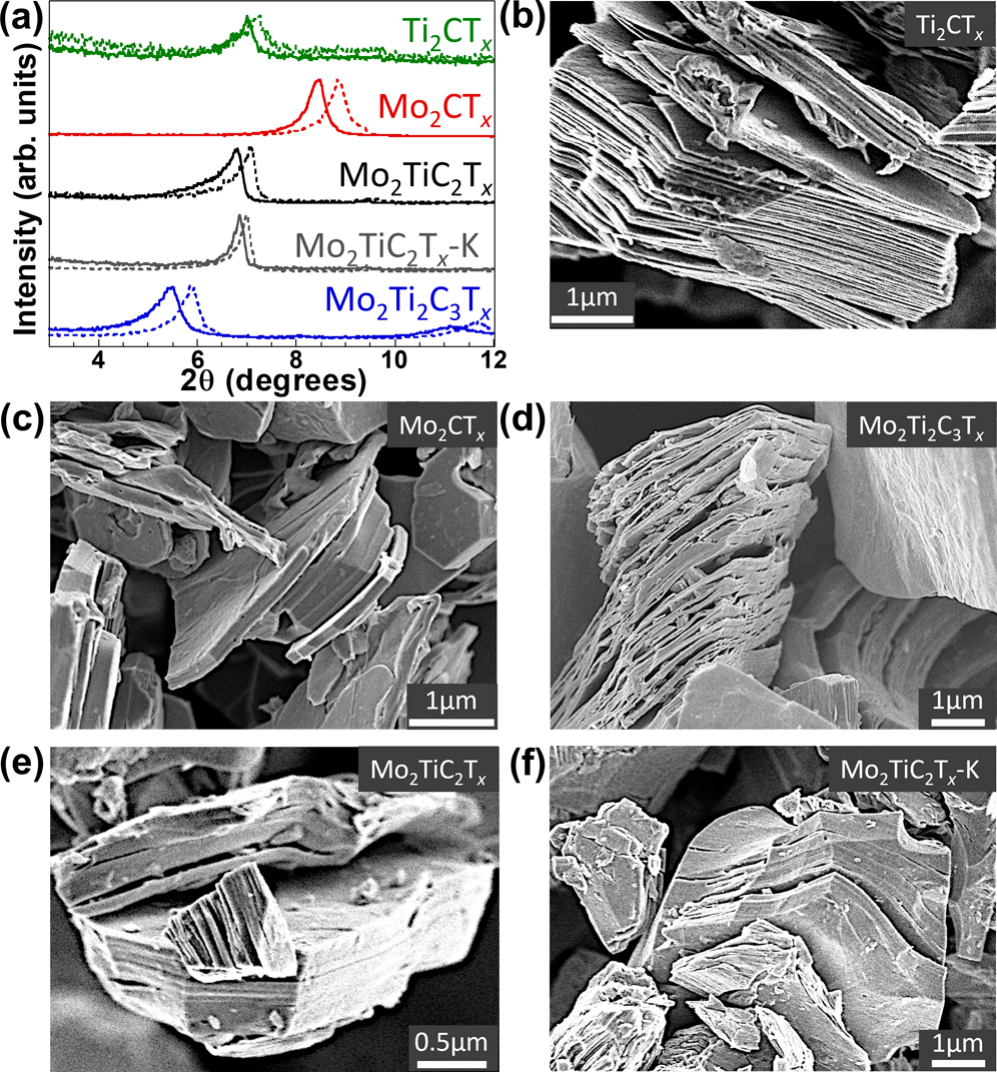
(a) XRD pattern showing (002) peaks before (solid lines) and after (dashed lines) vacuum annealing at 110 °C to remove bulk water. SEM images of (b)­Ti_2_CT_*x*_, (c) Mo_2_CT_*x*_, (d) Mo_2_Ti_2_C_3_T_*x*_, (e) Mo_2_TiC_2_T_*x*_, and (f) Mo_2_TiC_2_T_*x*_-K.

SEM images of the MXenes studied here are shown in Figure 2b–f. All exhibit morphology typical of MXenes, but there are notable subtle differences.^53^ While individual MXene layers (thickness of ∼10 Å) are not visible at SEM magnification, the longer-range morphology of the MXene multilayer stacks is visible. As shown in Figure 2b, Ti_2_CT_*x*_ displays the most open structure, with stacks of MXene flakes less than 10 nm thick and large slit-like pores of varying size between them. Conversely, the Mo- containing MXenes show a much more compact and platelet-like morphology, with the multilayer stacks reaching up to 1 μm thick and slit-like pores more sparsely distributed.

Inelastic neutron scattering was used to analyze the vibrational dynamics associated with MXene surface terminations. Figure 3 shows the INS results for the studied samples at various energies. It can be noticed that the Mo-containing MXenes are similar to Ti_3_C_2_T_*x*_ for most of the previously reported spectra.^19,36,54^ The bands below about 35 meV can be attributed to the translational vibrations of water. In the lowest energy range investigated (E_*i*_ = 55 meV), there is a small local maximum between 8 and 10 meV (Figure 3a), which can be attributed to bulk amorphous ice and surface water, as this peak corresponds to acoustic transverse modes in water molecules with a network of hydrogen bonds.^55^ The broad peaks between 40 and 140 meV (Figure 3b,c) correspond to librational motion in water molecules.^55^ The peak at 205 meV (Figure 3 c, d) can be assigned to intramolecular H–O–H bending vibrations, and the peak at ∼420 meV (Figure 3d) corresponds to O–H stretching modes in water, as has been observed before in bulk and confined water.^54,56^ All of these features are consistent with those previously reported for Ti_3_C_2_T_*x*_ MXene.^19^ Mo_2_TiC_2_T_*x*_–K shows the sharpest peaks for librational, bending, and stretching modes, suggesting that the water molecules are more ordered compared with other Mo-MXenes investigated here. This agrees with studies of K- intercalated Ti_3_C_2_T_*x*_, which show that water remains associated with the potassium ions.^51^ Also in Mo_2_TiC_2_T_*x*_–K, the librational band (50–100 meV, Figure 3b,c) is shifted to lower energy than in the other spectra, suggesting it displays the weakest hydrogen bonds, which again agrees with the presence of K^+^ disrupting the hydrogen bonding. The librational band for Mo_2_Ti_2_C_3_T_*x*_ is shifted to the highest energy, suggesting that it has the strongest hydrogen bonding.

**Figure 3:**
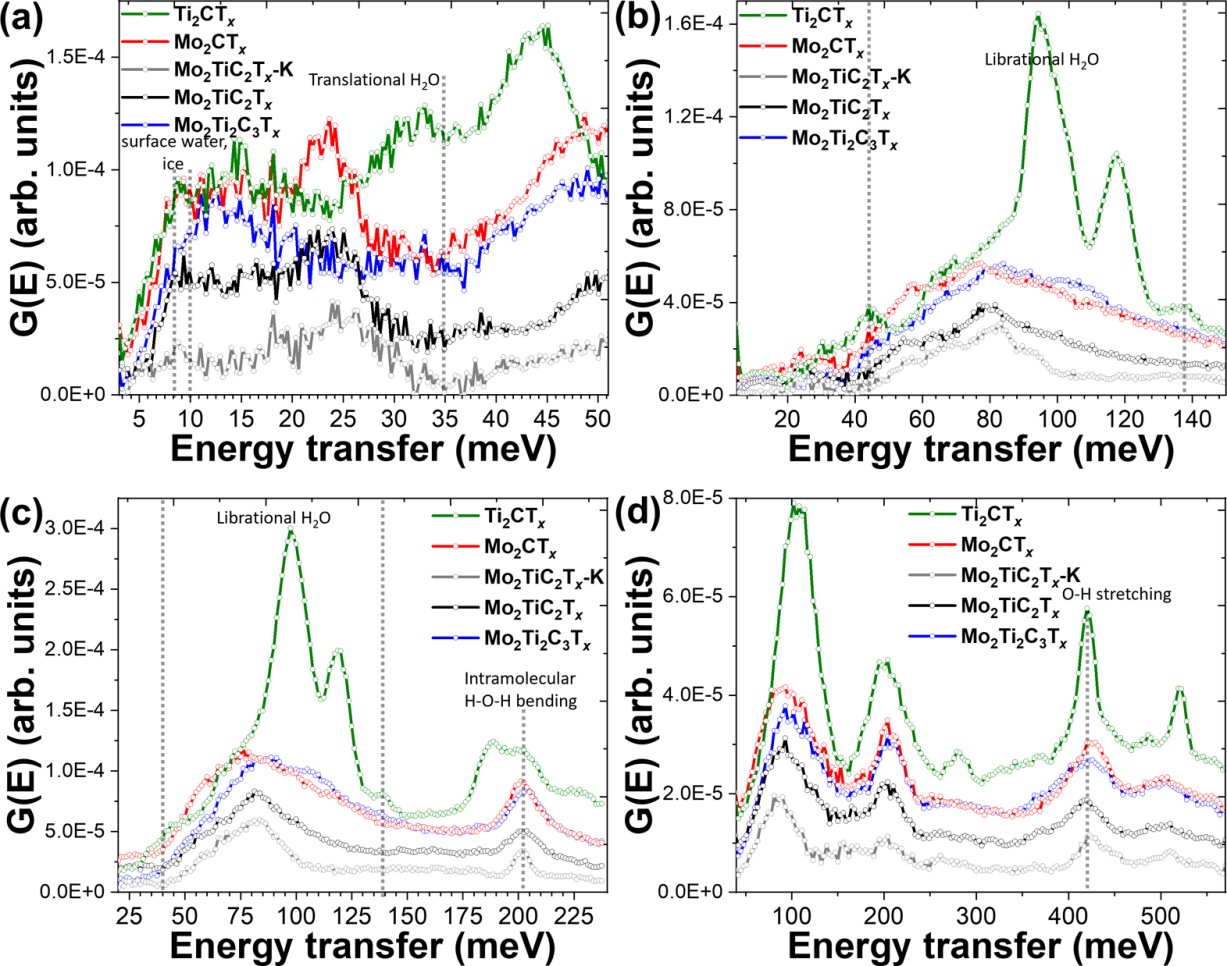
G­(E) spectra from inelastic neutron scattering of Ti_2_CT_*x*_ and Mo-containing MXenes at 5 K with incident energies of 55 (a), 160 (b), 250 (c), and 600 meV (d).

Unlike previous Ti_3_C_2_T_*x*_ MXene INS studies, Ti_2_CT_*x*_ does not show sharp peaks around 14.6 and 29.4 meV (Figure 3a), which had been attributed to trapped molecular hydrogen.^36,57^ Even more interestingly, Ti_2_CT_*x*_ shows a number of features that were not observed in the Ti_3_C_2_T_*x*_ and Mo-containing MXene system at all. As shown in Figure 4, when “baseline” MXene data (Mo_2_Ti_2_C_3_T_*x*_) are subtracted from Ti_2_CT_*x*_, thus removing the peaks that the two materials have in common, a few sharp, narrow, and large intensity peaks remain. Analysis of this difference spectrum can highlight the key differences between Ti_2_CT_*x*_ and other MXenes studied. The intramolecular water bending H–O–H peak observed in all spectra at ∼205 meV is not visible in the difference spectrum. That means that the difference spectrum does not relate to neutron scattering on water molecules. The two peaks at 95 and 118 meV are likely due to bending O–H hydroxyl groups perpendicular to the O–H bond, and a peak at 420 meV is due to the O–H stretching mode.^54,55,58,59^ The peaks between 160 and 240 meV, and between 260 and 380 meV can be respectively assigned to two- and three-phonon neutron scattering involving the modes at 95 and 118 meV.^60^ Similarly, the sharp peak at 520 meV can be attributed to a combination of the stretching (420 meV) and bending (95 meV) modes; the mismatch of (420 + 95 = 515 meV) is probably caused by anharmonicity.^60^ The presence of these extra peaks in the INS spectra of Ti_2_CT_*x*_, combined with their narrowness, indicates that Ti_2_CT_*x*_ contains more hydroxyl O–H groups than both the Mo-containing MXenes studied here and the Ti_3_C_2_T_*x*_ previously reported,^19,36^ and that these hydroxyl groups are well ordered.

**Figure 4:**
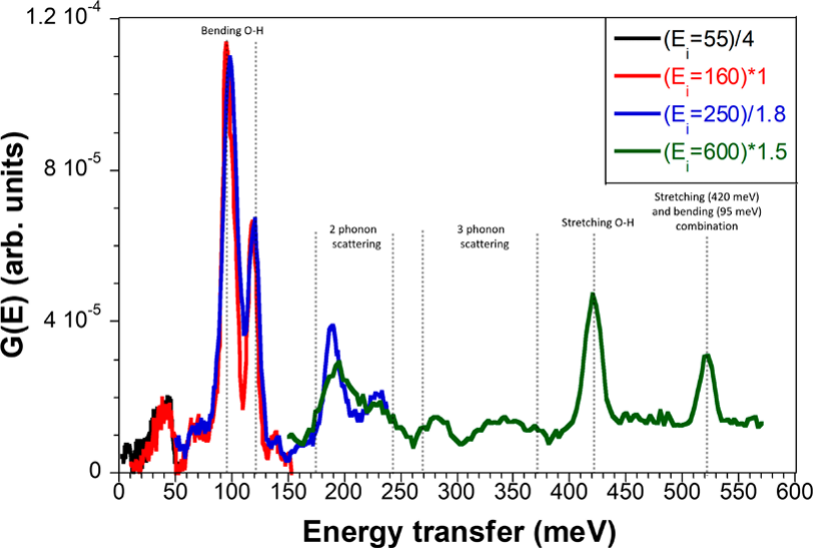
Difference spectra of Ti_2_CT_*x*_, generated by subtracting typical data from other measured MXenes (Mo_2_Ti_2_C_3_T_*x*_), followed by scaling to give a cohesive pattern obtained with different incident neutron energies (shown by different colors). Extra peaks for Ti_2_CT_*x*_ not found in other MXene samples are prominent and sharp.

To investigate the translational mobility of water molecules, the same samples were also studied using QENS. Since QENS relies on the incoherent scattering and hydrogen has by far the highest neutron incoherent scattering cross-section, the measured spectra are dominated by the contribution of the H atom present in the water molecules. Representative spectra (for *Q* = 0.7 Å^–1^) from all molybdenum-containing MXenes are shown in Figure 5 together with the fit obtained using a single Lorentzian function. Note that a single Lorentzian function was previously enough to capture the dynamics of confined water in pristine and metal ion intercalated MXenes.^18^ The mass-normalized elastic intensity at 20 K indicates the presence of more hydrogen in Mo_2_CT_*x*_ compared to other MXenes. Figure 6 shows a nonlinear Q^2^-dependence of the half width at half-maximum (HWHM) of the QENS spectra, suggesting a jump-type behavior of the confined water molecules.

**Figure 5:**
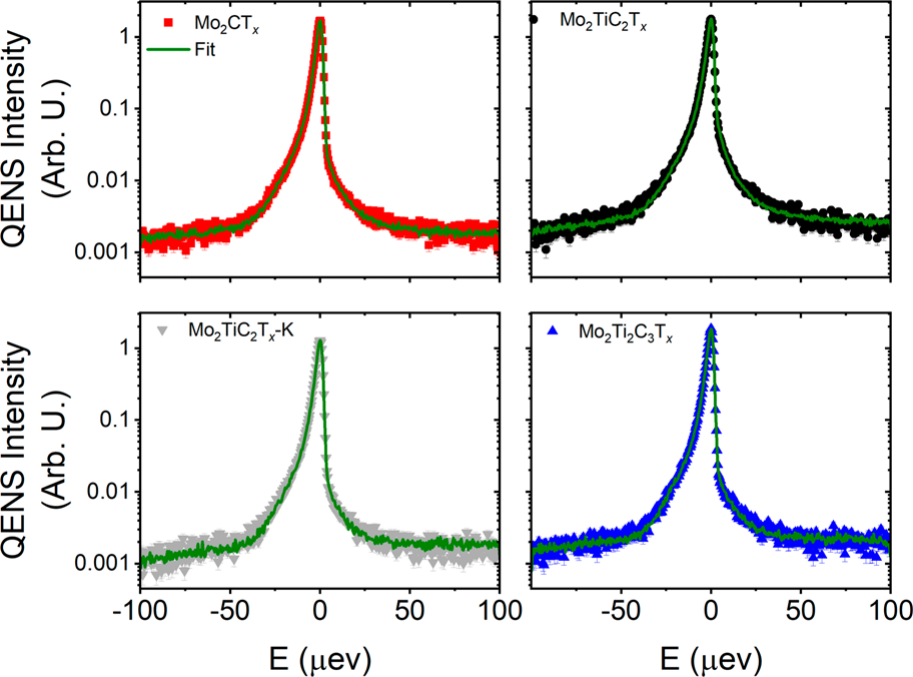
Representative QENS spectra (symbols) at *Q* = 0.7 Å^–1^ together with model fit (solid lines) for the molybdenum-containing MXene samples.

**Figure 6:**
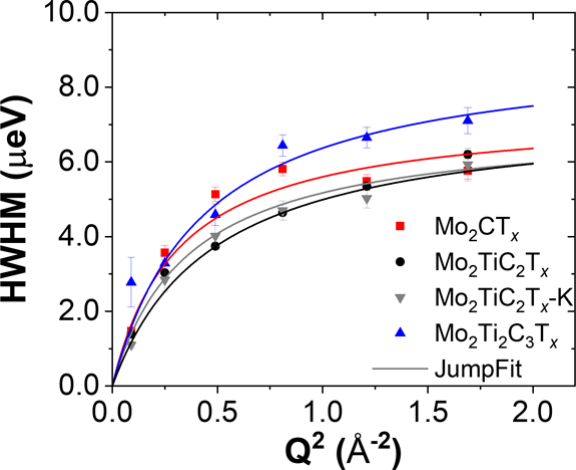
*Q* dependence of HWHM of QENS spectra obtained from a model described in the Supporting Information to determine the diffusion coefficients of water molecules.

Table 1 depicts the diffusion coefficients obtained from the jump diffusion model fit (eq 4 in the Supporting Information). The diffusion coefficients of the confined water are significantly smaller (by a factor of ∼8) compared to the bulk value,^61,62^ suggesting that the molecules are indeed confined. This is also evident from the absence of a sharp intensity drop in elastic scan profiles (for example, Figure 7b). Since the values are not that low compared to those of the bulk water, those water molecules are still not between the MXene layers. One would expect a huge reduction in the water diffusion coefficients if the molecules were confined between the layers.^18^ As in the case of the pristine Ti_3_C_2_T_*x*_, we anticipate that we are still probing the mobility of the water molecules residing in the gaps between the stacks of those molybdenum-based MXenes. Even though the diffusion coefficients for water molecules are fairly similar in all of the Mo-based MXenes, a higher diffusivity value in Mo_2_CT_*x*_ nicely corroborates the higher intensity observed on the mass-normalized QENS spectra due to the presence of more of the confined water molecules.

**Table 1:** Diffusion Coefficients of Water Obtained from QENS Data Analysis

	diffusion coefficient, D (10^–10^ m^2^s^–1^)	
samples	slow	fast
Ti_2_CT*_*x*_*	8.01 ± 3.4	23.2 ± 0.2
Mo_2_CT*_*x*_*	3.55 ± 0.5	
Mo_2_TiC_2_T*_*x*_*	2.94 ± 0.2	
Mo_2_TiC_2_T*_*x*_*-K	2.34 ± 0.2	
Mo_2_Ti_2_C_3_T*_*x*_*	3.21 ± 0.3	

**Figure 7:**
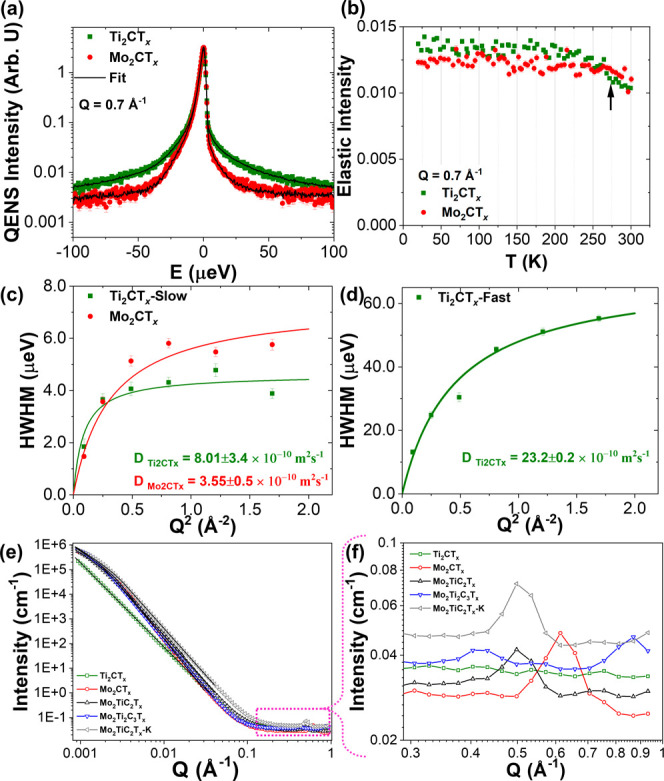
(a) Representative QENS spectra (for Q = 0.7 Å^–1^) measured at *T* = 300 K together with a model fit (solid line), (b) elastic scattering intensity as a function of temperature for Ti_2_CT_*x*_ and Mo_2_CT_*x*_ samples at the same Q, (c) *Q* dependence of HWHM of the QENS spectra analyzed with a jump diffusion model fit (solid line) to obtain diffusion coefficients of the slow component for water molecules in Ti_2_CT_*x*_ and Mo_2_CT_*x*_ (for the Mo_2_CT_*x*_ samples, it is the only component), and (d) shows the *Q* dependence for the fast component for water molecules in Ti_2_CT_*x*_. (e) SANS profile of all the samples together with Guinier-Porod model fits (solid line); (f) magnification of the marked region of panel (e), demonstrating high *Q* features in the samples. In the SANS data, the error bars were smaller than the markers and are therefore placed inside them, making them barely visible.

Ti_2_CT_*x*_ shows a behavior that is different from that of other MXenes studied. As shown in Figure 7a, the QENS signal measured from Ti_2_CT_*x*_ is much broader than the signal from Mo_2_CT_*x*_, which was the broadest among the Mo-containing MXenes. Indeed, a sum of two Lorentzians, one for slow and another for fast-moving water molecules, was needed to fit the QENS data for Ti_2_CT_*x*_ MXene. The necessity of the two-component model is also evident from a melting step observed around 275 K (indicated by an arrow in Figure 7b) in the elastic intensity scan profile, which, for example, is not present in Mo_2_CT_*x*_ MXene. Furthermore, the fit quality and the residuals resulting from a least-squares fit of the model to the data, presented in Figure S1, also support the necessity of a two-component model to fit the data. HWHMs of both components also show a strong nonlinear *Q* ^2^ dependence (Figure 7c,d). The diffusion coefficient obtained from the jump model for the slow component is 8.01 ± 3.4 × 10^–10^ m^2^ s^–1^, which is more than double the values for any of the molybdenum-containing MXenes but still lower than that of the bulk water, therefore corresponding to confined water. The fitting for the fast component (Figure 7d) gives a diffusion coefficient of 23.2 ± 0.2 × 10^–10^ m^2^ s^–1^, a value very close to the diffusivity of bulk water.^62,63^ It is the bulk-like water present in this sample that gives rise to the melting step indicated by the arrow in Figure 7b. It should be noted that, from the standpoint of QENS measurements and elastic intensity scans, bulk-like behaviors, such as a bulk water diffusivity value and melting exhibited at near zero °C, could still be associated with confined water, as long as its characteristic confinement size exceeds ca. 15–20 nm.^64,65^

The presence of two populations of water molecules in Ti_2_CT_*x*_ suggests the presence of heterogeneous morphology with a larger (>15–20 nm) and a narrower (<15–20 nm) interstack gap. It has been shown that the water confined in pores larger than 15–20 nm have diffusion coefficient similar to that of the bulk water.^64,65^ The SANS profile (Figure 7e) of Ti_2_CT_*x*_ does not show finite peaks either in low or intermediate *Q*, suggesting the presence of totally uncorrelated structures that might provide a morphology with gaps of different dimensions. This agrees with the SEM images shown in Figure 2b, where Ti_2_CT_*x*_ showed thin sections of MXene with variable slit pores between them. To further confirm these results, mercury porosimetry measurements were performed on Ti_2_CT_*x*_ and Mo_2_CT_*x*_. Ti_2_CT_*x*_ (Figure S2a) exhibits a higher volume fraction of larger pores, including a substantial volume fraction of pores in the 1–2 μm range, a noticeable number of pores in the 15–20 nm range, and almost no pores smaller than 15 nm. In contrast, Mo_2_CT_*x*_ (Figure S2b) shows, in addition to a large volume fraction in between 0.2 and 1 μm, a significantly smaller fraction of pores in the 15–20 nm range compared to Ti_2_CT_*x*_. Moreover, the presence of sub-10 nm pores can be observed for Mo_2_CT_*x*_. Given that mercury porosimetry typically struggles to resolve pores in the sub-10 nm size regime, the presence of a measurable fraction in this range is particularly notable. Although the total pore volume associated with these pores is relatively low, their high number density is sufficient to produce the signal observed in Figure S2b. Overall, the porosimetry data further support the conclusions drawn from the QENS and SANS results. A sharp upturn at low *Q* together with a Porod exponent of 3.4 from the Guinier-Porod fit reflects the presence of large structures with rough surfaces.^66^ On the other hand, Mo-containing MXenes show the leveling off of the SANS intensity in low *Q* (*Q* ≈ 0.025 Å^–1^) due to the existence of larger size assembled layered stacked structures with a thickness of ∼100 nm (Table 2) and of smooth surfaces. Again, this observation in the SANS results agrees with the SEM in Figure 2c–f, where the stack size in the Mo-containing MXenes is significantly larger than that observed for Ti_2_CT_*x*_, with many stacks on the order of ∼100 nm. The peaks observed in high *Q* (Figure 7f) are indications of layered structures, where the *d*-spacings obtained from the *Q* positions of the Bragg peaks (*d* = 2π/*Q*) match nicely with the *d*-spacings obtained from XRD.

**Table 2:** Parameters Obtained from SANS Data Analysis Using a Guinier–Porod Model

	low *Q* Guinier–Porod fit			high *Q*, peak position
samples	radius of gyration, *R*_g_ (Å)	thickness, T (Å)	Porod exponent	interlayer spacing, Å
Ti_2_CT*_*x*_*			3.4	10.2 12.5 14.7 (1st), 7.3 (2nd order) 12.4
Mo_2_CT*_*x*_*	328.5	1137	4.3	
Mo_2_TiC_2_T*_*x*_*	339.5	1176	3.8	
Mo_2_TiC_2_T*_*x*_*-K	274.0	949	3.8	
Mo_2_Ti_2_C_3_T*_*x*_*	400.4	1387	4.0	

Combining the results of the three neutron studies, it is clear that only subtle differences exist for most MXenes in terms of the structure and dynamics of confined water and in terms of the long-range structure of the MXene layer stacks. As showin in Figure 8, Ti_2_CT_*x*_ stands out as an outlier. This difference in behavior can be caused by several factors. Etching of Ti_2_CT_*x*_ can be challenging because it reacts strongly with the HF etchant, requiring 10% HF rather than 50%, and the reaction is very brief, needing only 2 h to etch rather than 24+ h for the other MXenes. Further, there is considerable weight loss for this MXene, especially if some of the MAX phase particles are small. For this study, MAX phase particles between −200 and +325 mesh (44–74 μm) were selected for Ti_2_CT_*x*_ as compared to −400 mesh (37 μm or less) for the other Mo-containing MAX phases. These factors combined suggest that Ti_2_CT_*x*_ is more likely to have more defects as compared to the other MXenes studied. The difference in particle size could also contribute to the differences in the layer stack size. In addition to defects caused by etching and possible particle size differences, the molecular weight of Ti_2_C­(OH)_2_ (−OH has been used as the only termination for simplicity of calculation) is the lowest of all the MXenes studied here, and in fact, it is the smallest of all MXenes. Its molecular weight is only about 70% that of Ti_3_C_2_(OH)_2_, whereas the Mo-MXenes are about 118% (Mo_2_C­(OH)_2_), 148% (Mo_2_TiC_2_(OH)_2_), and 178% (Mo_2_Ti_2_C_3_(OH)_2_) of the mass of Ti_3_C_2_(OH)_2_. Thus, for Ti_2_CT_*x*_, the surface terminations represent a higher percentage of the mass than in the other MXenes and can have a much larger impact on the structure and properties.

**Figure 8:**
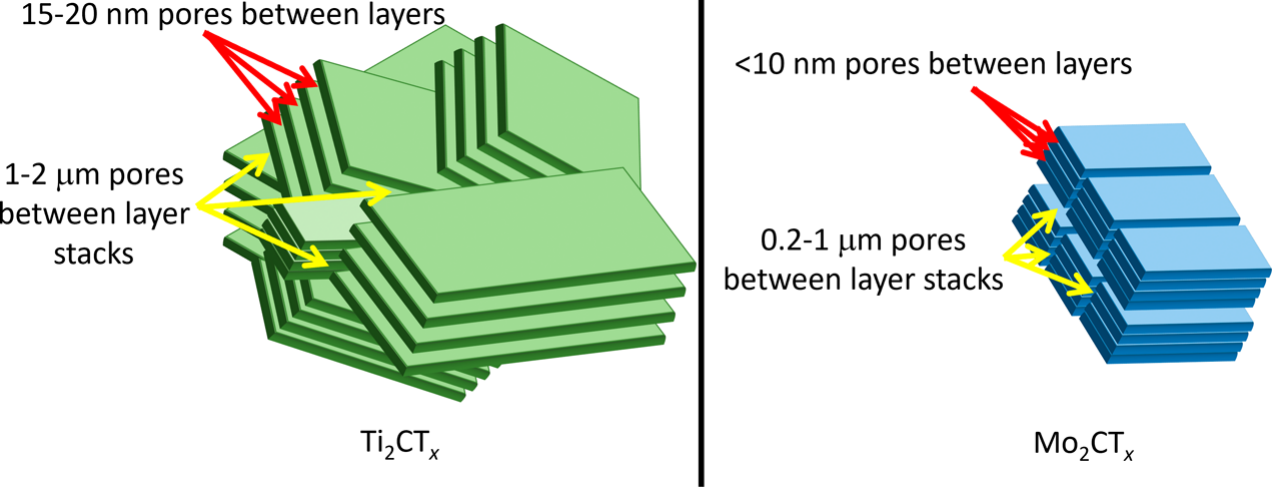
Schematic showing the differences in porosity and morphology, as indicated by QENS, SANS, and mercury porosimetry. Mo_2_CT_*x*_ is shown here because it was used for the porosimetry measurements, but the conclusions apply to all of the Mo-containing MXenes.

## Conclusions

Neutron studies of MXenes with structures and compositions different from Ti_3_C_2_T_*x*_—both in terms of layer thickness “*n*” and in terms of transition metal—show that, for the most part, these variations did not significantly alter the trends observed and previously reported for Ti_3_C_2_T_*x*_. Indeed, based on these data sets, only small structure-dependent changes are observed. For the Mo-MXene system, Mo_2_Ti_2_C_3_T_*x*_ (*n* = 3) exhibited the strongest hydrogen bonding, whereas Mo_2_CT_*x*_ (*n* = 1) displayed the highest mobility, with Mo_2_TiC_2_T_*x*_ (*n* = 2) in the middle. However, we did see an exception for Ti_2_CT_*x*_, which demonstrated several key differences across all of the techniques studied, even when compared with existing results for Ti_3_C_2_T_*x*_. These differences were evident even in routine analyses, such as XRD and SEM. XRD revealed a larger interlayer spacing, suggesting either a higher content of intercalants or weaker hydrogen bonding in Ti_2_CT_*x*_. SEM images showed that Ti_2_CT_*x*_ forms thinner layer stacks with more slit pores compared to the Mo-containing MXenes. INS measurements show additional peaks for Ti_2_CT_*x*_, which are consistent with Ti_2_CT_*x*_ possessing higher hydroxyl group contents than in Mo-containing MXenes or in the previously reported Ti_3_C_2_T_*x*_ MXenes. QENS further highlights distinct behavior observed in Ti_2_CT_*x*_, requiring a two-component fit and revealing two populations of water, indicative of variable interstack gaps. The SANS data further displayed differences between Ti_2_CT_*x*_ and other MXenes studied, also confirming the presence of gaps with varying dimensions. These observations point to differences in the long-range structure and suggest that Ti_2_CT_*x*_ possesses a higher concentration of well-ordered hydroxyl surface terminations.

Such differences may arise from multiple factors, including variations in the particle size, etching conditions, and intrinsic structural distinctions within the MXene family. Further studies are needed, however, to fully understand the nature of Ti_2_CT_*x*_ surface terminations and the reasons behind its unique characteristics compared to other MXenes. Overall, this study enhances our understanding of the behavior of water in confinement with MXene layers, which can guide the development of MXenes for electrochemical applications such as energy storage and electrochemical water purification.

## Supplementary Material



## Notes

Data reduction and analysis details for INS, QENS, and SANS, a comparison of the QENS one- and two-component fits for Ti_2_CT_*x*_, and pore distribution graphs for Ti_2_CT_*x*_ and Mo_2_CT_*x*_ (PDF)
