# Supplementary material for: Structure and Dynamics of Water Confined in Transition Metal Carbide MXenes: Implications for Electrochemical Applications
Source: ACS Appl Nano Mater. 2026 Jan 29;9(6):2862–71. doi: 10.1021/acsanm.5c05156 (PMC12910551; doi:10.1021/acsanm.5c05156)
Supplement: Supplementary file 1 [file an5c05156_si_001.pdf]

Supporting Information

Structure and Dynamics of Water Confined in Transition Metal Carbide MXenes: Implications for Electrochemical Applications

Kaitlyn Prenger[1], Alexander I. Kolesnikov[2], Naresh C. Osti[2], Eugene Mamontov[2], Jong K. Keum[2,3], Kenneth C. Littrell[4], and Michael Naguib[1*]

[1]Department of Physics and Engineering Physics, Tulane University, New Orleans, LA 70118, USA

[2]Neutron Scattering Division, Oak Ridge National Laboratory, Oak Ridge, TN 37831-6473, USA

[3]Center for Nanophase Materials Sciences, Oak Ridge National Laboratory, Oak Ridge, TN 37831, USA

[4]Neutron Technologies Division, Oak Ridge National Laboratory, Oak Ridge, TN 37831, USA

* naguib@tulane.edu

Details of Data Analysis for Neutron Scattering Techniques

INS:

The collected neutron scattering data were transformed from time-of-flight and instrument coordinates to the dynamical structure factor $S(Q,E)$ using the software package mantidplot[1] and then to a generalized vibrational density of states

$$G(E) = S(Q,E)\, E/\{Q^2[n(E,T)+1]\} \quad (1)$$

where $n(E,T) = 1/[\exp(E/k_BT)-1]$ is the population Bose factor.

QENS:

In QENS, the overall measured scattering intensity, $I(Q,E)$, is given by[2]

$$I(Q,E) = [X_1(Q)\delta(E) + (1 - X_1(Q))S(Q,E) + B(Q,E)] \otimes R(Q,E) \quad (2)$$

In equation 2, the fraction of elastic scatting contribution is accounted for by $X_1(Q)$, whereas the delta function, $\delta(E)$, captures the intensity at zero energy transfer in reference to the instrument resolution width. It is the dynamic structure factor, $S(Q,E)$, which contains the information regarding the motion of water molecules confined in the samples. $B(Q,E)$ is a linear background term added to account for contribution from various sources. The data comprising the elastic and quasielastic scattering terms in equation 2 is convoluted during the data analysis to the instrument resolution, $R(Q, E)$, measured from the very same sample at

cryogenic temperatures, when the scattering signal becomes purely elastic. The $S(Q,E)$ was modeled to the Lorentzian functions:

$$S\,(Q,E) = \frac{1}{\pi}\frac{\Gamma_i(Q)}{\Gamma_i^2(Q)+E^2} \quad (3)$$

Here, $\Gamma_i(Q)$ is the half width at half maximum (HWHM) of Lorentzian functions where the subscript *i* can be 1 or 2 or even 3 depending on the number of relaxation processes present in a sample. Out of five MXenes samples, only the QENS data collected from Ti_2C MXene needed two Lorentzians to fit the data. The width of a Lorentzian function can be used to extract the diffusion coefficient of water molecules using a jump diffusion model as

$$\Gamma_i(Q) = \frac{DQ^2}{1+DQ^2\tau_o} \quad (4)$$

where D and $\tau_o$are the diffusion coefficient and residence time of the water molecules.

SANS

Low-Q ($Q <$ ~0.04 Å^{-1}) SANS data were fit using the generalized Guinier-Porod model as follows,[3]

$$I(Q) = \frac{G}{Q^s} exp\left(\frac{-Q^2R_g^2}{3-s}\right) for\ Q \le Q_1\ (Guinier\ term) \quad (5)$$

$$I(Q) = \frac{D}{Q^m} for\ Q \ge Q_1\ (Porod\ term) \quad (6)$$

where I(Q) is the scattered intensity, Q is the scattering variable, R_g is the radius of gyration, *m* is the Porod exponent, and *G* and *D* are the Guinier and Porod scale factors, respectively. For the Guinier and Porod terms to be continuous at Q_1, the following relations are derived:

$$Q_1 = R_g^{-1}\left(\frac{3m}{2}\right)^{\frac{1}{2}} \quad (7)$$

$$D = G\ exp\left(\frac{-Q_1^2R_g^2}{3}\right)Q_1^m = G\ exp\left(-\frac{m}{2}\right)\left(\frac{3d}{2}\right)^{m/2}\frac{1}{R_g^m} \quad (8)$$

For three dimensional globular objects such as spheres, rods and platelets (or lamellae), s = 0, 1 2, respectively. R_g's for spheres of radius of R, randomly oriented rods of cross-sectional radius, R and randomly oriented platelets of thickness are given by $R_g = R\sqrt{3/5},\ T/\sqrt{12}$, respectively. Also, for a Porod exponent m = 4 points to particles with smooth surfaces while m = 3 points to very rough surfaces. In the current study, $s \cong 2$ and the thickness of platelets T were obtained as $T = \sqrt{12}R_g$.

Comparison of Single Lorentzian and double Lorentzian fits for Ti_2CT_x

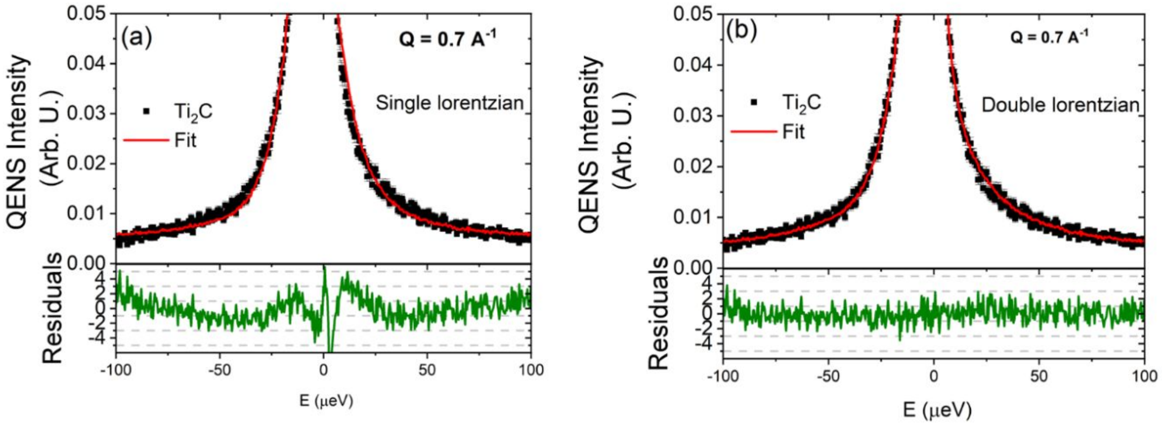

Figure S1. A representative fit of the QENS data from Ti_2CT_x at Q = 0.7 $Å^{-1}$: (a) single Lorentzian and (b) double Lorentzian models. Top panels show the experimental data with the corresponding model fits, while bottom panels display the residuals.

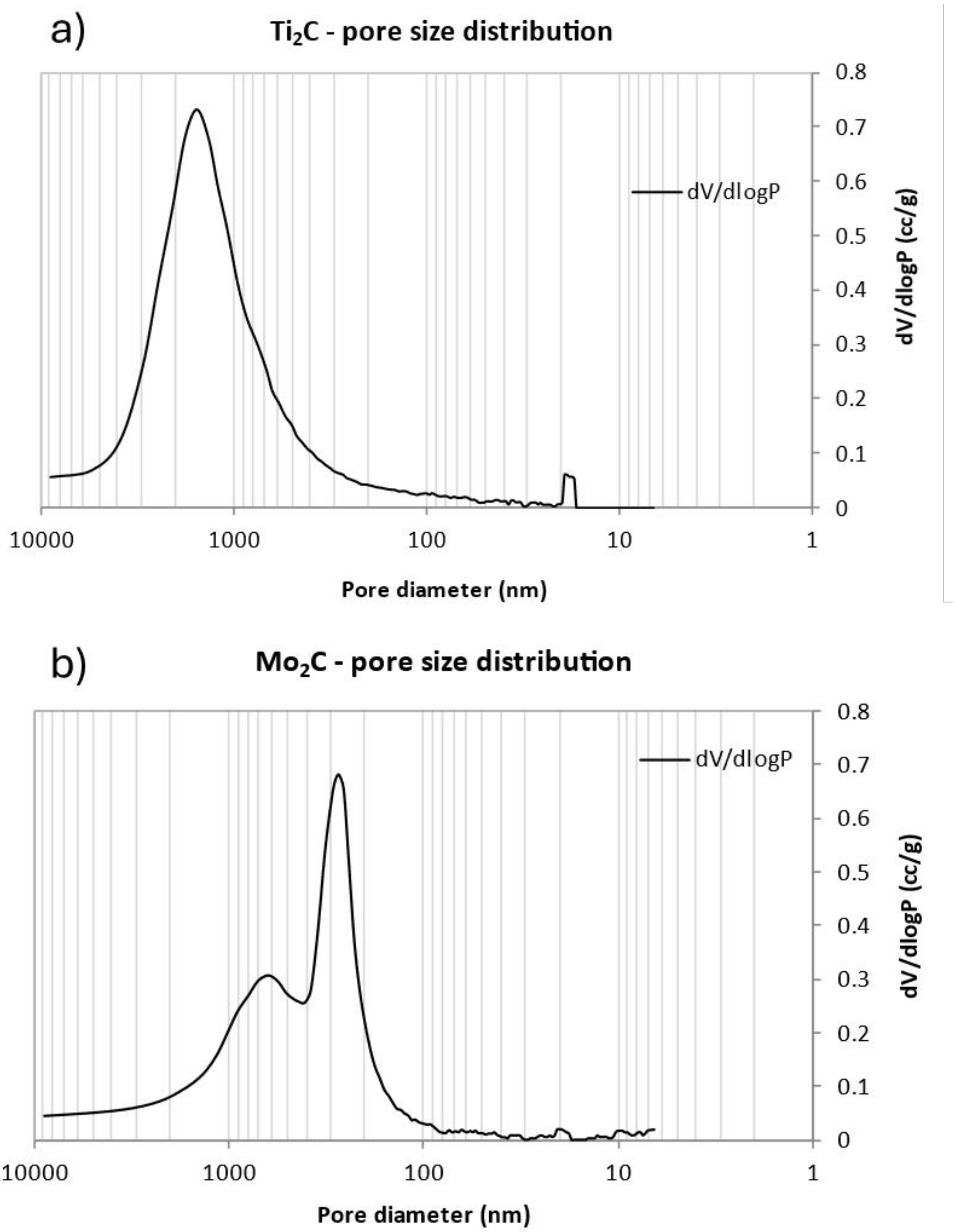

Figure S2 Pore size distribution for(a) Ti_2CT_x and (b) Mo_2CT_x from mercury porosimetry.

References:

(1) Arnold, O.; Bilheux, J. C.; Borreguero, J. M.; Buts, A.; Campbell, S. I.; Chapon, L.; Doucet, M.; Draper, N.; Ferraz Leal, R.; Gigg, M. A.; et al. Mantid—Data analysis and visualization package for neutron scattering and μ SR experiments. *Nucl. Instrum. Methods Phys. Res. A* **2014**, *764*, 156-166. DOI: https://doi.org/10.1016/j.nima.2014.07.029.
(2) Bee, M. *Quasielastic Neutron Scattering: Principles and Applications in Solid State Chemistry, Biology, and Materials Science*; Adam Hilger, Bristol, 1988.
(3) Hammouda, B. A new Guinier-Porod model. *J. Appl. Crystallogr.* **2010**, *43*, 716-719. DOI: 10.1107/s0021889810015773.
